# Point Cloud Fusion of Human Respiratory Motion Under Multi-View Time-of-Flight Camera System: Voxelization Method Using 2D Voxel Block Index

**DOI:** 10.3390/s25103062

**Published:** 2025-05-13

**Authors:** Jiadun Wang, Shengtao Li, Kai Huang

**Affiliations:** 1 School of Computer Electronics and Information, Guangxi University, Nanning 530004, China; 2113391065@st.gxu.edu.cn; 2 School of Computer Science and Engineering, Sun Yat-sen University, Guangzhou 510006, China; lisht7@mail2.sysu.edu.cn

**Keywords:** time-of-flight camera, multi-view point cloud fusion, multi-camera system, voxelized scenes, respiratory motion point cloud, surface reconstruction

## Abstract

Time-of-flight (ToF) 3D cameras can obtain a real-time point cloud of human respiratory motion in medical robot scenes. Through this point cloud, real-time displacement information can be provided for the medical robot to avoid the robot injuring the human body during the operation due to the positioning deviation. However, multi-camera deployments face a conflict between spatial coverage and measurement accuracy due to the limitations of different types of ToF modulation. To address this, we design a multi-camera acquisition system incorporating different modulation schemes and propose a multi-view voxelized point cloud fusion algorithm utilizing a two-dimensional voxel block index table. Our algorithm first constructs a voxelized scene from multi-view depth maps. Then, the two-dimensional voxel block index table estimates and reconstructs overlapping regions across views. Experimental results demonstrate that fusing multi-view point clouds from low-precision 3D cameras achieves accuracy comparable to high-precision systems while maintaining the extensive spatial coverage of multi-view configurations.

## 1. Introduction

With the rapid development of medical robots, high-precision real-time monitoring of human thoracic and abdominal respiratory motion has become a core requirement to improve the performance of surgical navigation systems. A multi-camera 3D point cloud acquisition system based on the principle of time of flight (ToF) [1] can be applied to the field of respiratory motion monitoring. Compared with the single-camera system, the components of the multi-camera system can compensate each other through information from different perspectives to obtain more accurate three-dimensional information.

At present, time-of-flight cameras mainly adopt two modulation schemes: amplitude-modulated continuous-wave (AMCW) modulation and Pulse-Based (PB) modulation [2,3], which are referred to as AMCW-ToF and PB-ToF in this paper. AMCW-ToF cameras achieve measurements through phase difference measurements with higher static accuracy, but generate mutual interference in the case of multiple cameras; PB-ToF cameras do not generate mutual interference, but under the current technical conditions, where the camera can be used in the case of multiple cameras, the accuracy of this kind of camera is poor, making it difficult to meet the accuracy requirements of a surgical robot for respiratory motion monitoring.

Given the current hardware constraints, there is a contradiction between larger coverage and higher accuracy when obtaining the point cloud of the chest and abdomen, which is a very difficult topic; in particular, the medical robot scene also needs to have higher real-time requirements. In response to these challenges, a multi-camera acquisition system is designed and built in this paper. The GPIO interface of Raspberry Pi is used to control the opening and closing sequence of the camera, and the depth frames of multiple perspectives with appropriate time intervals are obtained. After obtaining the depth data of multiple views, we develop a multi-view fusion algorithm of human thorax and abdomen point clouds based on a 2D voxel block index, and through this algorithm, the depth data of the thorax and abdomen can be obtained; the depth accuracy of the pulse-wave ToF camera with lower accuracy is improved to be close to that of the CW-ToF camera with higher accuracy.

The following are the main contributions of this study:

1. A three-view acquisition system combining an AMCW-ToF camera and a PB-ToF camera is designed and built. The AMCW-ToF camera is used to provide a high-precision reference point cloud, and the PB-ToF camera suitable for multi-view is used as the input of the algorithm to solve the problem of the lack of point cloud samples of human respiratory motion. In addition, a more economical 3D camera is used, avoiding the high cost of building a high-precision positioning system.
2. Based on the demand of multi-view fusion, a two-dimensional index table based on voxel blocks is proposed to manage voxel blocks of different views by constructing corresponding voxel blocks for different views; the isolation between voxels of different views is realized to avoid access conflicts. At the same time, the corresponding voxel storage table is constructed to store voxel blocks of different views, and this voxel storage table does not need to store all voxels in the voxel block, but only needs to store the observed depth data.
3. In the face of the collision and overlap of voxel blocks from different perspectives, this paper is inspired by the best suture theory in image registration and designs an algorithm to estimate the overlapping area based on the two-dimensional index table of voxel blocks; according to the position information of voxel blocks from different views in a unified view, the algorithm finds the overlapping area of two views by vertical alignment and horizontal alignment, and the algorithm avoids the discontinuity of the overlapping region obtained by the conventional collision detection algorithm.
4. Using the orderliness of the points stored in the voxel block in the voxel block two-dimensional index table and the similarity of the point clouds from different perspectives, we solve the problem of point cloud redundancy and stratification in the overlapping region of the two perspectives to solve the problem of point cloud completion using known points; a point cloud completion algorithm for overlapping regions is designed to achieve fast and effective overlapping region repair.

We collected the respiratory motion data of ten volunteers on the completed three-view acquisition system and constructed a human respiratory motion dataset, which is compared with the commonly used point cloud registration and point cloud smoothing algorithms. The experimental results show that our algorithm can break through the hardware limitation of the low-precision 3D camera, and the accuracy of the obtained point cloud is close to that of the higher-precision 3D camera by fusing the information of multiple perspectives.

This paper is organized as follows. In Section 2, we describe the related research. In Section 3, we introduce the hardware platform and the data acquisition process. In Section 4, the method proposed in this paper is elaborated, and the assessment results of the method are presented in Section 5. Section 6 is the discussion. Section 7 summarizes this paper, and proposes the future direction of improvement.

## 2. Related Works

In this section, the background and related research of point cloud reconstruction of human respiratory motion are described, mainly from two directions: the transition of respiratory motion information and 3D cameras, and 3D reconstruction algorithms.

### 2.1. Respiratory Motion Information and 3D Cameras

The earliest use of respiratory motion information was in medical imaging [4] and radiotherapy. Obtaining the respiratory motion information of patients can allow accurate judgement of imaging and avoid imaging artifacts caused by respiratory motion. The movement of tumors can be estimated by respiratory motion information [5], and radiotherapy can be performed at the right time to avoid damage to normal tissues. With the development of 3D cameras, the respiratory motion data on the patient’s body surface can be easily obtained by using non-contact sensors. Three-dimensional cameras, which can capture three-dimensional information through binocular imaging, structured light, and time-of-flight methods, have advantages in terms of cost and hardware ease of use. The time-of-flight [6] (ToF)-based 3D camera has certain advantages and the lowest computational complexity in the face of human skin, which is a relatively single-color surface. Therefore, the ToF camera is very suitable for the reconstruction of human respiratory point clouds. In the research of using ToF cameras to obtain the point cloud of human respiratory motion, part of the research  [7,8,9,10,11,12] is to use ToF to classify respiratory motion and measure respiratory depth.

In a study similar to the purpose of this paper, Ref. [13] studied the extraction of a respiratory motion surface with boundary markers and compared it with the data of respiratory flow meter, and Refs. [14,15] extracted respiratory motion, and tumor markers in patients were jointly estimated. In these studies, only a single ToF camera was used, and the coverage of the chest and abdomen provided by a single camera is limited, making it difficult to deal with some scenarios that require surgery from the side. Of course, some studies have used multiple ToF cameras, such as study [16] using two cameras, but the frame rate of a single camera is only seven frames, which is obviously not enough for medical robots.

### 2.2. Real-Time 3D Reconstruction Algorithms

The hardware used in real-time 3D reconstruction algorithms is the same as ours, and their research purpose is partly similar to that of this paper. The main difference lies in the difference between the reconstruction objects and the reconstruction results. Early algorithms for reconstructing a static scene in real time using a 3D camera can be traced back to Kinect-Fusion [17,18], a classic scene reconstruction algorithm that reads a depth stream through a handheld 3D camera; the static scene is reconstructed based on a TSDF voxelized scene and ICP registration algorithm. In the follow-up research, there is InfiniTAM [19], a large-scale static scene reconstruction algorithm that uses voxel coordinates to map to hash values to save the memory overhead of voxelization and the method of saving memory by using Sparse Voxel Octree [20] to compress voxel expression. In the reconstruction of dynamic scenes, Richard A, in his Dynamic-Fusion [21], pioneeringly uses volumetric 6D motion fields to represent the real-time deformation of objects, using multiple cameras to capture the dynamic actions of the human body; however, these studies are often used in VR and AR, as well as SLAM, which are different from the point cloud reconstruction of the human chest and abdomen in real time in medical scenes.

## 3. Data Acquisition

### 3.1. Multi-View 3D Camera System Architecture

A multi-view 3D camera system was established to meet the data acquisition requirements. The three viewpoints were arranged symmetrically around the geometric center of the supine subject’s thoracoabdominal region: one directly above the target area and two laterally positioned at left/right oblique angles. Each viewpoint comprised paired Vzense D560C [22] (Gore Microelectronics Co., Ltd., Qingdao, China) and Intel Realsense L515 [23] (Intel Corporation, Santa Clara, CA, USA) cameras (six units total), with the D560C employing pulse-wave modulation and the L515 utilizing continuous-wave modulation technology. Cameras within each viewpoint were aligned collinearly, maintaining a lateral separation of 25 cm to prevent mutual field-of-view occlusion while preserving depth measurement accuracy through a forward-facing orientation. All cameras were rigidly mounted on aluminum extrusion frames, ensuring mechanical stability during prolonged experiments.

Each view of the camera system is connected to a computer equipped with a high-speed solid-state drive for fast data storage through a corresponding interface. In the software, the camera is controlled and the data are stored by a specially developed acquisition program, and the information of the timestamp is stored at the same time. We also provide an external electrical signal through the Raspberry Pi to the model D560 camera for more accurate control.

**Figure 1:**
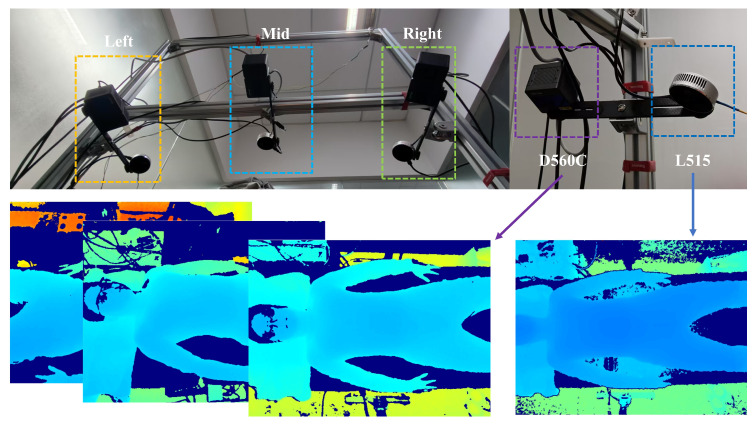
Three-view multi-camera acquisition system with two cameras per view.

After the construction is completed, to compare the point clouds obtained from different views in the same coordinate system, it is necessary to calibrate the cameras to determine the transformation matrix between them. The multi-camera acquisition system is shown in Figure 1.

### 3.2. Acquisition Settings and Volunteer Information

In this paper, we used the method of recruiting volunteers to obtain data on the human thorax and abdomen. A total of 10 volunteers were recruited, all male. These volunteers were in good health and had no abnormal conditions affecting respiratory movement at the time of experimental data collection. The volunteers were dressed in grey bodysuits and lay on their backs, breathing evenly. In addition, we recorded the volunteers’ physical information, as shown in Table 1.

In the acquisition system, the external input signal of D560C is set to enter the high-level signal according to the order of “left, right, and middle” in each cycle, and the duration of the high-level signal is 0.5 milliseconds and the duration of the low level is 14.5 milliseconds, with a total period of 600, which in theory yields a total of 1800 depth frames. Both the D560C and L515 depth frames are set at 640 × 480 resolution and do not capture RGB streams, which would not capture the volunteers’ faces.

After the setup is complete, for each volunteer, perform the following data collection procedure:

1. Turn on the L515 camera in one direction and wait for a second before turning on the D560C camera in the left, middle, and right.
2. Wait until each view in the D560C camera has acquired 600 depth frames, and then stop all camera acquisition.
3. Repeat steps 1–2 for each direction, ensuring that multiple-view depth maps are available for each view as experimental data and for comparison.

In the data collected in this section, the depth map of D560C will be used as input to the algorithm in this paper, and the depth map obtained by L515 will be used as a comparison, due to the higher depth accuracy of the L515 camera, but it cannot be acquired at the same time (multi-camera interference of CW modulation camera), so it is used as the contrast object.

For each volunteer, we recorded only their height, weight, and age, and used a code name to represent the corresponding data. We did not record any information that uniquely identified the individual, and the data collection process did not have any harm or impact the volunteers. In accordance with relevant provisions, this study can be exempted from ethical review. Prior to the collection of the human data, we informed the volunteers of the purpose of the experiment and their rights and risks, and obtained their informed consent.

## 4. Materials and Methods

**Figure 2:**
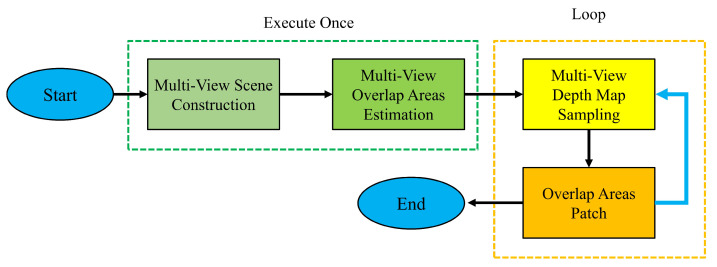
An overview of the proposed method.

This section describes our voxelization-based multi-view depth data fusion algorithm. The algorithm designs a data structure supporting a multi-view voxelized scene, which can construct the scene through the depth of data under multiple views. After constructing the scene, the overlapping regions of voxel blocks in different views are obtained by the algorithm similar to the best suture line in the image domain. In the face of the overlapping region, we design a point cloud completion algorithm to deal with the fusion problem of points from different perspectives in the overlapping region and finally obtain a high-quality multi-view fusion point cloud of the human chest and abdomen. The overall flow of the algorithm is shown in Figure 2.

### 4.1. Multi-View Scene Construction

**Figure 3:**
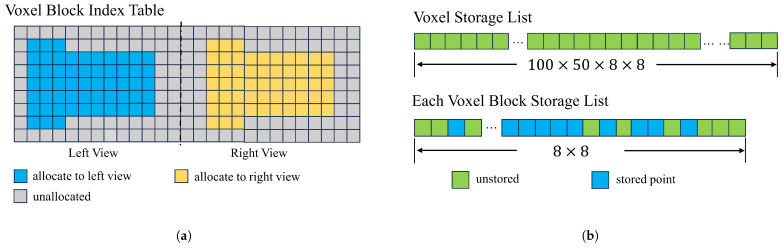
Voxel block index table and voxel storage list. (**a**) Voxel block index table, where each index element is assigned to a different view. (**b**) Each voxel is stored in a list that identifies which information has been stored.

To facilitate data access and reduce conflicts between views, a two-dimensional voxel block index table is designed to manage voxel blocks of different views and the actual storage area of voxel blocks of different views; our voxel block borrows from the implementation of voxel blocks in InfiniTAM [24]. These two structures are called voxel block index table and voxel storage list. These two data structures are shown in Figure 3.

The voxel block index table shown in Figure 3a is a two-dimensional table that stores information about voxel blocks, indicating whether elements are assigned to voxel blocks and whether the area of the depth map needs to be assigned to voxel blocks. In our design, different regions divided by columns will be assigned to voxel blocks in different views to avoid storage conflicts. For example, with a voxel size of 5 mm, the left and right views can be fully covered by pre-assigning a two-dimensional table with a length of 100 and a height of 50. Columns 1 through 50 are assigned to the left view and columns 51 through 100 are assigned to the right-hand view.

The voxel storage list shown in Figure 3b is a linear table of custom voxel structures, where each element stores a three-dimensional piece of information. In other studies [19], individual voxels require less storage space (minimum 4 bytes; our 12 bytes) but have a larger number of constituent voxel blocks. In the process of voxelizing the depth information of the chest and abdomen, only some voxels can be used. Therefore, we have improved the storage of voxel blocks from storing all the voxels of a voxel block (which requires the allocation of the length of L×W×H) to storing all the pixels of a voxel block, improved to storing only the valid voxels on each vertical column of the voxel block (only the length of L×W needs to be allocated); this improvement reduces the memory required for voxel storage by 96%.

When constructing the scene, you need to enter a depth map from a different perspective. Firstly, the thoracic and abdominal regions are extracted through the corresponding mask, which can avoid the interference of other regions. Secondly, for each depth map, the depth value of the effective region is traversed, and the depth value D(u,v) stored by the pixel is converted to the $Point_{cam}$ under the current camera coordinate by the camera internal parameter *K*. Finally, the $Point_{cam}$ is converted into the voxel coordinate, and the voxel coordinates in a certain range are converted into the voxel block coordinate $voxelBlockPos(v_{x},v_{y},v_{z})$ utilizing interception. Geometrically, the meaning of this operation is to divide the points in a certain region of space into the range of the voxel block. Formula (1) describes this process, where *L* is the side length of the voxel block.

(1) $$\begin{array}{c} voxelBlockPos\left(v_{x},v_{y},v_{z}\right)=\left\lfloor \frac{Point_{cam}}{voxelSize\times L}\right\rfloor \end{array}$$

After obtaining the coordinates of each voxel block, each voxel block is stored in the voxel block index table for easy access. The table index (Row, Col) is calculated by adding the distance on the negative half-axis, as shown in the Formula (2), where offset is the column offset, which in the experiment is set to half the width of the voxel index table.

(2) $$\begin{array}{cc} row^{left} & =\left(v_{x}^{l},\;v_{y}^{l},\;v_{z}^{l}\right)+h_{neg}^{left}+1,\\ col^{left} & =\left(v_{x}^{l},\;v_{y}^{l},\;v_{z}^{l}\right)+w_{neg}^{left}+1,\\ row^{right} & =\left(v_{x}^{r},\;v_{y}^{r},\;v_{z}^{r}\right)+h_{neg}^{right}+1,\\ col^{right} & =\left(v_{x}^{r},\;v_{y}^{r},\;v_{z}^{r}\right)+w_{neg}^{right}+1+offset. \end{array}$$

**Figure 4:**
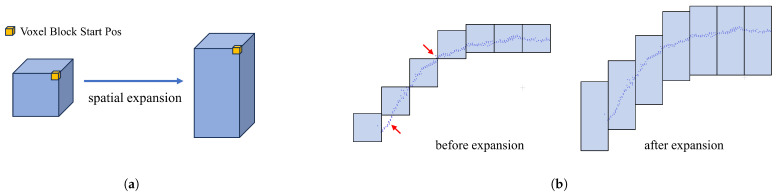
Enlarging voxel block size to avoid missing regions. (**a**) The height of the voxel block is set to three times the original height. (**b**) The point pointed at by the red arrow is not covered by the voxel block before stretching, while the voxel block after stretching is completely covered.

In addition, in the experiment of constructing the scene, we found that for the depth data of the human chest and abdomen with a certain curvature, missing areas that could not be covered by voxel blocks (see red arrows in Figure 4b) appeared in some areas. This problem is probably due to type conversion during the interception of voxel block coordinates.

To solve the problem of this missing region, we solve it by optimizing the *z*-axis coverage of voxel blocks. Specifically, the general voxel block size is composed of 8 × 8 × 8 voxels, whose starting and ending coordinates are $StartPos(v_{x},v_{y},v_{z})$ and $EndPos(v_{x},v_{y},v_{z}+8)$, respectively, near and far from the camera along the *z*-axis, and decrease and increase the size of a voxel block, respectively, so that in this section, the voxel block is sized 8 × 8 × 24 with $StartPos(v_{x},v_{y},v_{z}-8)$ and $EndPos(v_{x},v_{y},v_{z}+16)$ coordinates. Geometrically, the cube-shaped voxel block is elongated into a column-shaped voxel block. The operation increases the coverage of each voxel block in the positive and negative directions along the *z*-axis; these designs can avoid missing depth samples in subsequent procedures, as shown in Figure 4.

### 4.2. Multi-View Overlap Area Estimation

In the fusion of multi-view voxelized scenes, the obtained depth information is often redundant, especially in our scene, and the point clouds of chest and abdomen from different views will have a large overlap area. Therefore, overlapping regions are estimated in this section to determine which voxel blocks are overlapping, which voxel blocks need to be retained, and which are not required prior to the sampling step.

An easy way to think of this is to unify the voxel blocks of the left and right side views into the middle view through the rotation and translation matrix RT and treat the voxel block as a cuboid, through the three-dimensional AABB [25] collision box algorithm, to determine whether they overlap. We initially experimented with this idea but found that the voxel blocks obtained by the algorithm were not continuous with each other in the same perspective, and there were more independent voxel blocks clustered together. Our guess is that there are more overlapping regions than independent regions in this scene.

**Figure 5:**
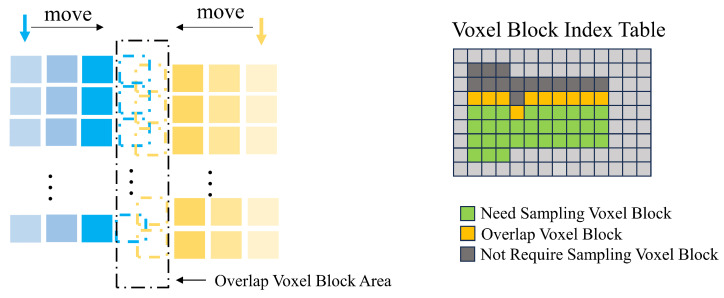
The overlapping regions of voxel blocks from different views are estimated by the algorithm in this section.

In this study, we are inspired by the best suture algorithm [26] in the image domain and apply the idea of this algorithm to voxel blocks of multi-view. In this algorithm, the index table of voxel blocks is regarded as 2D images taken from different perspectives, and the position information of voxel blocks stored in the index is used as the basis for judging whether there is a overlap. The steps of the algorithm are as follows. Figure 5 shows the process of finding overlapping voxel blocks and labeled voxel blocks.

First, set the indices pointing to the voxel table in two directions, $L_{i},L_{j}$ and $R_{i},R_{j}$. *i* corresponds to the row of the table and *j* corresponds to the column of the table. Because voxel blocks from different views are positioned differently in the index table when they overlap, we need to set the range of $L_{j}$ to (1,50), $R_{j}$ to (100,51), and $L_{i}$$R_{i}$ both to (50,1).

Second, align the block of the starting voxel. The coordinates of voxel blocks in different directions can be obtained by the subscript mentioned earlier. This integer coordinate $Pos_{voxelBlock}$ can be converted into the coordinates $Pos_{cam}$ in the camera coordinate system and then unified into the coordinates $Pos_{world}$ in the world coordinate system by the rotation and translation matrix. At this time, the distance between the two coordinates on the *x*-axis in the world coordinate system is judged. If the distance is less than the threshold *T* (*T* = voxelSize × 8), the starting voxel block meets the requirements. If the distance is greater than the side length of the voxel block, then move the Index J in one direction (fixing $L_{j}$ in this section, moving $R_{j}$) until a block of eligible voxels is found.

Third, after the second step, align the starting voxel blocks in the two directions, and the overlapping region of the voxel block can be obtained by the algorithm in this step. In the voxel block index table, the overlapping voxel blocks will be marked. Algorithm 1 shows the detailed process of this step.

For more detailed logic, we used pseudocode, see Algorithm 1.
**Algorithm 1:** Multi-view overlap area estimation algorithm
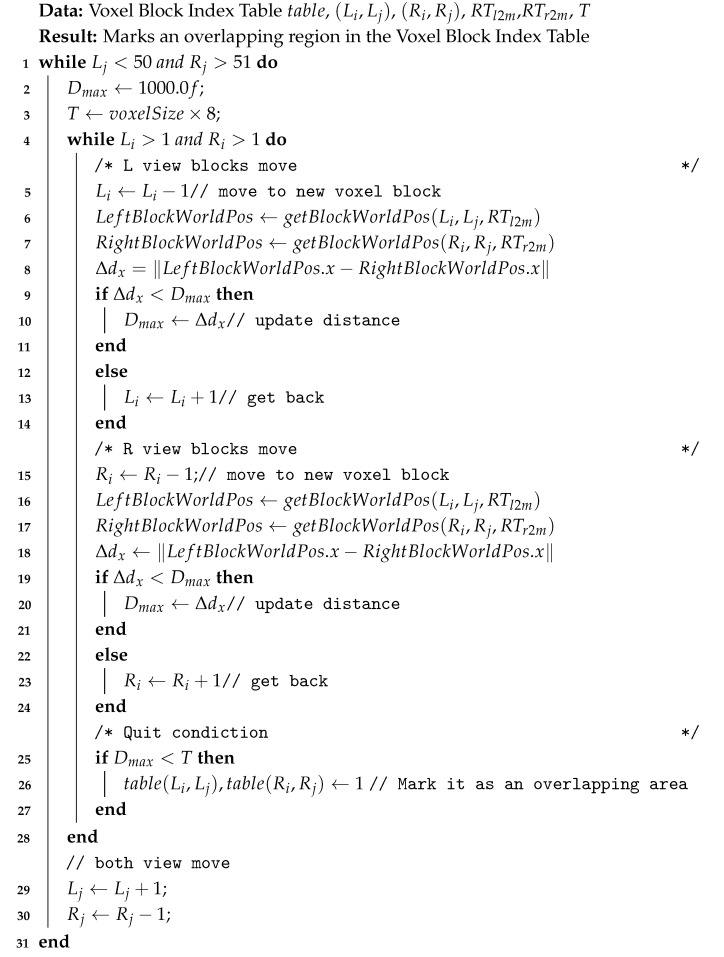


   When the third step finds the collision voxel block, the column belonging to the vertical direction is moved, and then the horizontal movement alignment process is repeated. Finally, the existing valid voxel blocks are traversed to complete the search of the overlapping region of voxel blocks. While identifying the overlapping voxel blocks, the other voxel blocks are marked, and all the valid voxel blocks in the positive direction of the row index of the overlapping voxel block are set to the voxel blocks that need to be sampled; all valid voxel blocks in the negative direction are set to voxel blocks that do not require sampling.

### 4.3. Multi-View Depth Map Sampling

**Figure 6:**
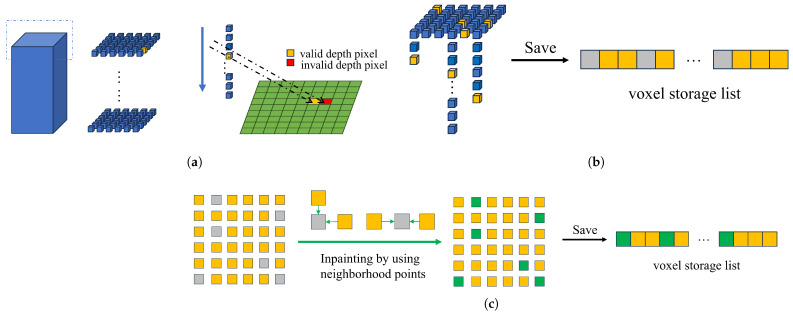
The process of sampling a depth map from a single voxel block. (**a**) Each voxel is traversed first according to the *z*-axis and projected into the depth map to find the depth value. (**b**) A voxel with a depth value found in the voxel block will be stored in the voxel storage table. (**c**) Using neighborhood points to repair the missing points in voxel blocks.

In this section, sampling is performed through voxel blocks identified as normally sampled in the voxel block index table, as shown in Figure 6 below. The voxel block is a cube or cuboid made up of voxels measuring 8 × 8 × 24. Each voxel is a small cube with a side length of 5 mm.

As shown in Figure 6, (a) corresponds to finding valid pixels, (b) to store 3D points, and (c) to missing 3D point inpaint. These three steps are described in detail below.

Finding valid pixels: This algorithm traverses each voxel in the voxel block and converts the coordinate information of the voxel pos into a 3D point in the camera’s view; then, a virtual depth value $d_{v}$ is obtained by projecting the intrinsic parameter matrix *K* of each camera to the depth map corresponding to each viewing angle. If there is a valid pixel value in the pixel coordinate corresponding to $d_{v}$, the proximity between the depth pixel in the pixel coordinate and the depth value of the projection is judged. If the proximity is within the allowable range, a reasonable pixel value is found. This allowable range is set to the size of the side of a voxel, which is 5 mm.

Store 3D points: When the depth value is found, the coordinates of the current 3D point and the corresponding depth value are stored in the voxel storage list. In contrast to how the entire voxel block is stored in other studies (requiring L×W×H), our storage area only uses a continuous space of length L×W in the voxel storage list, which is used to store the entire voxel block; the voxels in the voxel block that successfully obtain the depth value are stored in the region using the row-first method. Under different views, the following Formula (3) is also used to obtain the starting position of the continuous region to avoid the storage conflict between different voxel blocks.

(3) $$\begin{array}{cc} Addr_{L} & =\left(Li\times W_{table}+L_{j}\right)\times L_{block}\times W_{block}\\ Addr_{R} & =\left(Ri\times W_{table}+R_{j}\right)\times L_{block}\times W_{block} \end{array}$$

Missing 3D Point Inpaint: When a voxel block sampling is completed, due to the sampling process involving several different data type conversions, there will be some situations where pixel values are missing. To this end, we add an inpainting step after sampling each voxel block. By accessing the valid points in the four-neighborhood, the points in the four-neighborhood are completed according to the three-dimensional information of the valid points.

### 4.4. Overlap Area Patch

After sampling multiple depth maps from different perspectives, the depth information of the corresponding region is obtained for each normally sampled voxel block. At this time, it is necessary to process the points identified as overlapping voxel blocks to finally produce a more complete human thorax and abdomen point cloud.

In a single voxel block, the original discrete 3D points can be quickly accessed by the local index of each voxel block. At the same time, 3D cameras with different viewing angles should capture the same points in the same area as close as possible in space during similar shooting times. Therefore, we transform the processing goal of the points in the overlapping region into a point completion algorithm for the overlapping region; through the similarity of the points close to each other from different perspectives and the similarity of the points in the overlapping region, the algorithm of this section is designed, and the neighborhood points can be accessed directly by the local index of voxel blocks.

**Figure 7:**
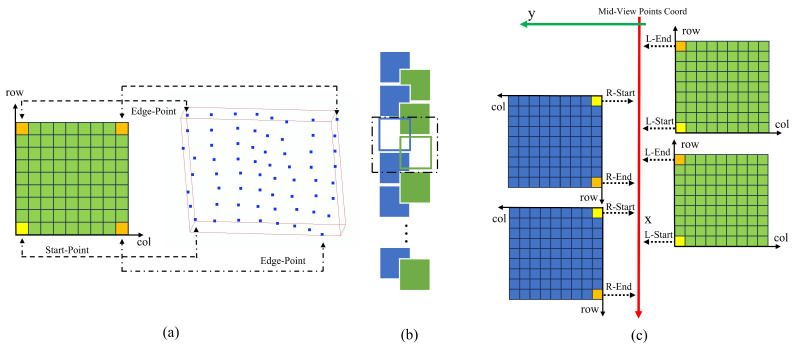
Repair process. (**a**) Voxel blocks store 3D points that can be positioned directly using row and column indexes. (**b**) The sliding window method processes two voxel blocks at a time. (**c**) The voxel blocks of different views are aligned according to the starting point and the end point, and point completion is performed.

**Figure 8:**
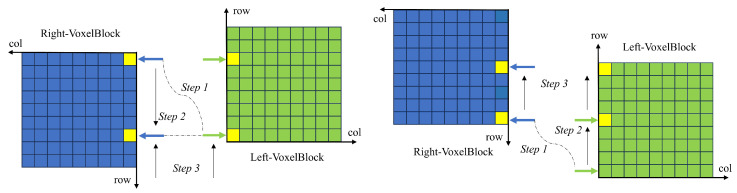
Cursor movement in two overlapping cases.

The algorithm processes the points stored in the horizontally adjacent voxel blocks of the overlapping area and generates new points to fill the overlapping area by using the position information of the points near the center line in the two directions. The algorithm consists of the following steps:

1. The voxel blocks in the overlapping region of different views are used as the processing objects, and the sliding window method is used to process two voxel blocks at a time, which are derived from different views; there can be left–right and right–left combinations.
2. For these two voxel blocks with different views, their boundary points are extracted by the index to determine their position and overlap relationship. The definition of boundary points is shown in Figure 7a. A voxel block stores up to 4 valid boundary points. In this section, just two of them, the end and start points on the *x*-axis projection, are needed, and the different views are captured in slightly different ways.
3. After obtaining the start point and end point of the different views, determine whether the point pairs of the different views overlap. In the ideal case, the projection of the *x*-axis from a uniform perspective, where S for L corresponds to E for R, and E for L corresponds to E for R, is unusual. In practice, the voxel blocks of l-view and r-view are partially overlapped with each other, and often overlap with two voxel blocks in the other direction. Thus, the criterion for the overlap of voxel blocks in the two directions is set to *L-Start > R-End* and *R-Start > L-End*, corresponding to L blocks in front of R blocks and L blocks behind R blocks, respectively, as shown in Figure 7b.
4. When it is determined that two voxel blocks overlap on the *x*-axis, the subscripts of one view are moved, and the stored points corresponding to the subscripts of different views are aligned on the *x*-axis. In the case where LR overlaps, as shown in Figure 7c, two cursors in the LR direction are first initialized, both pointing to the starting point, and the distance between the starting points is obtained and converted to the number of rows moved by the formula, and the r-direction cursor is moved to the new position. The cursors in the L and R directions point to the two closest points on the *x*-axis; again, this is the case when the LR directions overlap, as in Figure 8. The difference is that the first initialized cursor has R pointing to the end point and L pointing to the start point. When moving the cursor, move L’s cursor to the new position.
5. After the alignment in Step 4, the points pointed by the cursors in the different directions are closest on the *x*-axis. At this point, calculate the distance between two points on the *y*-axis; according to the size of the voxel needed to fill the number of point steps, calculate the difference between two points △(x,y,z), and through Formula (4), a new point is generated by the difference between the component and the step length *k*.
  (4) $$\begin{array}{cc} N & =\left\lfloor \frac{D_{hrozi}}{voxelSize}\right\rfloor \\ Points_{new} & =Point_{start}+▵\left(x,y,z\right)*k\\ k & =1,2,3,\ldots ,N \end{array}$$
6. Repeat steps 3 through 5 to complete the completion of the overlapping region adjacent to the current two voxel blocks. After completing this pair of voxel blocks, the sliding window moves one step to continue processing another combined voxel block and finally completes all the tasks.

The concrete process of the algorithm in this section can also refer to Algorithms A1 and A2 in Appendix A.

Figure 8 depicts how the cursor moves in two overlapping cases, corresponding to steps 4–5 of the above.

In Figure 8, *Step 1* computes the vertical distance from the point on the starting line, *Step 2* converts the vertical distance to the number of steps moved, and *Step 3* completes the code by moving the cursor.

## 5. Experiments and Results

**Figure 9:**
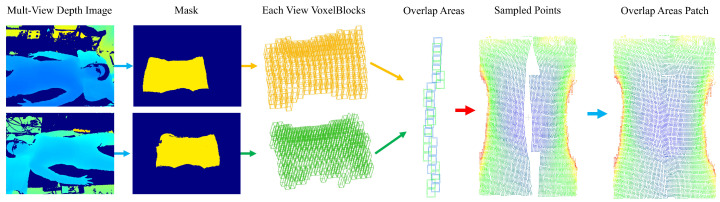
Visualization of the experimental process.

Using the respiratory motion data of 10 volunteers obtained in the data acquisition phase, this paper implements the algorithm in C + + on a computer equipped with AMD Ryzen 3600 CPU. In order to evaluate the effect of the algorithm in this paper, we compare the point cloud acquired by the L515 camera under the same view angle as the ground truth (GT) during the data acquisition phase. Figure 9 shows the process of obtaining the fused human thorax and abdomen point cloud by a series of calculations with the data from the two-view depth map of D560C.

### 5.1. Evaluation Metric Selection

To quantitatively evaluate the multi-view fusion effect of the proposed algorithm, the following two directions are selected for quantization:

Spatial Similarity Metrics: Chamfer Distance (CD) and Hausdorff Distance Distance (HD) are two classical metrics in point cloud quality assessment, both of which can quantify the similarity of spatial distribution between 3D point sets. The difference is that the Hausdorff distance is less robust to the Shandong of the local noise point, whereas the Shandong distance is more robust to the local noise point. Considering the relatively small number of outliers in the point cloud, the CD distance thatis more suitable for this situation is selected for evaluation.

Temporal Consistency Metrics: According to the data characteristics of this paper and the experience in the field of respiratory motion, we need to quantify the results of this algorithm in time and judge whether the point cloud output of the fusion algorithm can reflect the actual respiratory motion of the patient.

### 5.2. Spatial Similarity Results

The contrast data selected in this paper are the 3D camera data collected synchronously from L515, and the depth accuracy of the 3D camera is higher than that of D560C. In comparison, the data of L515 are divided into three parts according to different perspectives, and the data of D560C are aligned in time and space, and their CD values are calculated as indicators. In the algorithm settings, the voxel sizes are set to 5 mm and 3 mm.

In terms of comparison methods, we chose Gauss smoothing [27] and moving least squares (MLS) [28] for comparison. Both methods can achieve the purpose of smoothing the point cloud and significantly improve the quality of the point cloud.

Table 2 demonstrates consistent improvements in geometric accuracy (measured by Chamfer Distance, CD) across all test cases. Our voxel block-based multi-view fusion algorithm reduces the CD between fused and ground-truth (GT) point clouds by up to 39.72% (average 20.08%) at a 5 mm voxel size, and 60.91% (average 22.31%) at a 3 mm voxel size. In comparison with other methods, the error of the proposed algorithm is better than that of other methods in 90% of the samples.

**Figure 10:**
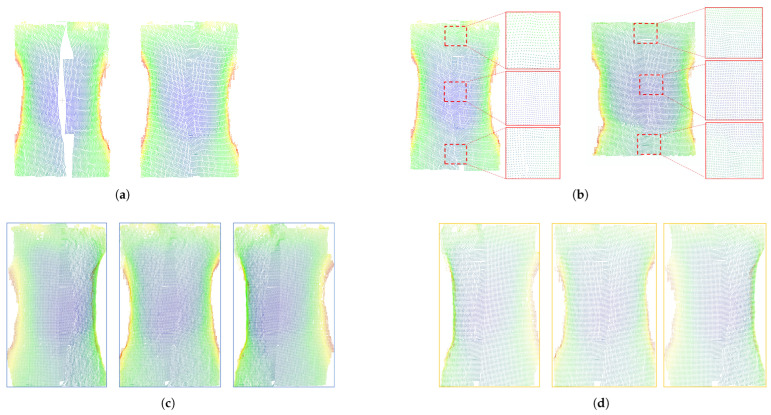
The point cloud display obtained by the algorithm fusion in this paper. (**a**) Left- and right-side point clouds and fused point clouds; (**b**) detail of point cloud overlapping region after patch; (**c**) 5 mm voxel based point cloud; (**d**) 3 mm voxel based point cloud.

**Figure A1:**
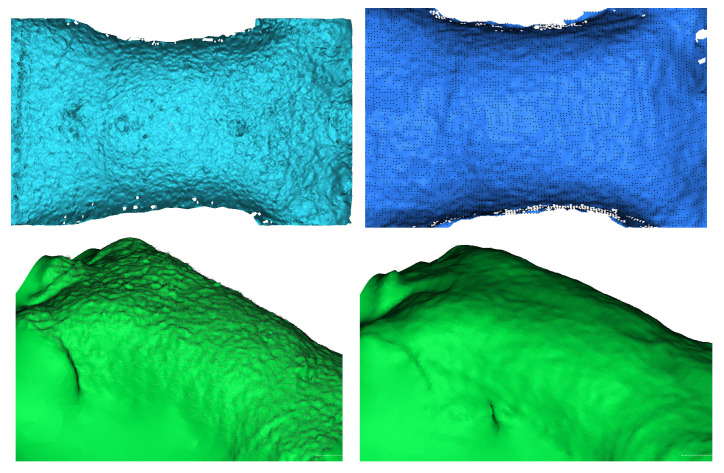
The left side is the triangulation of the D560C camera point cloud, and the right side is the triangulation of the result of this algorithm.

**Figure A2:**
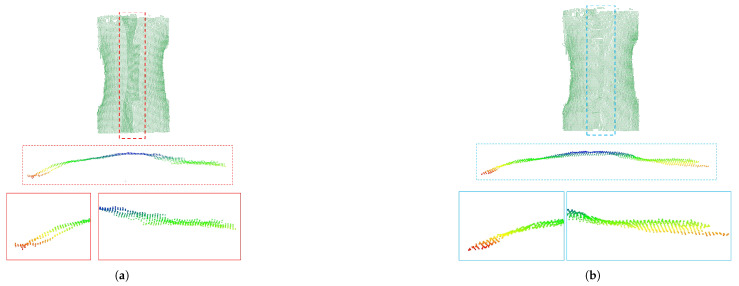
The overlap areas processed by the algorithm in this paper (cross-sectional view). (**a**) Unprocessed overlapping region point cloud (**b**) The point cloud of the overlapping region processed by the algorithm in this paper.

As shown in Figure 10, the algorithm in this paper can fuse the point clouds from different perspectives through voxelized scenes to generate a high-quality point cloud with an effect close to that of a higher-precision ToF camera. In addition, we carried out Delaunay triangulation of the point cloud based on the results of this algorithm, and the results can be found in Appendix Figure A1. Appendix Figure A2 shows the point cloud after Overlap Area Patch processing.

### 5.3. Time Consistency Results

This section presents a comparison of respiratory motion curves under dynamic conditions. The choice of the curve is to find the average depth of the point cloud centroid. Consecutive frames numbered 100 to 500 were selected, starting at frame 100, to avoid short-term respiratory fluctuations caused by the volunteer hearing the experimenter’s instructions before the data acquisition. In addition, we also quantitatively study the contrast results between the point cloud of respiratory motion obtained by the original D560C camera and the point cloud generated by the fusion algorithm, see Table 3.

**Figure 11:**
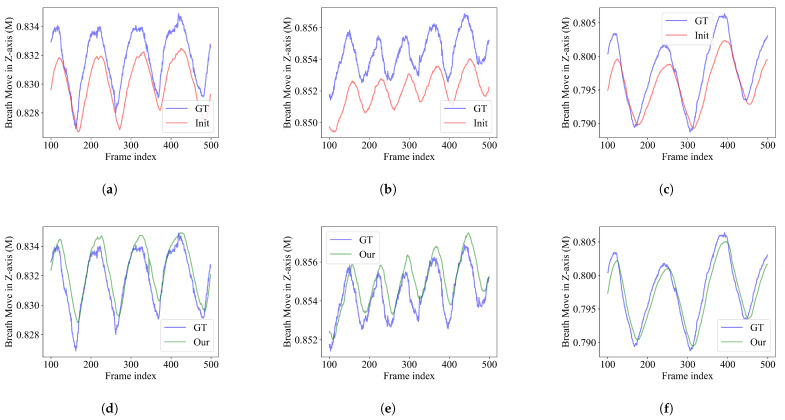
Waveform of respiratory movement in volunteers with different numbers. (**a**–**c**) The breathing motion of a volunteer, as reflected in point clouds from the D560C and L515 cameras, respectively. (**d**–**f**) Represents the data of a volunteer, the results of our algorithm, and the respiratory motion reflected in the point cloud from the L515 camera.

As shown in Figure 11, above is the respiratory motion waveform captured by the original D560C camera and the L515 camera in the middle view, and below is the respiratory motion waveform between the results of this algorithm and the L515 camera. It can be seen that the point cloud obtained by the algorithm in this paper shows higher consistency in the respiratory waveform. In the quantitative analysis shown in Table 3, 10 of the volunteers improved their Pearson product–moment correlation coefficient scores, and 7 of them improved their scores to near 1; the lowest coefficient in the initial comparison, 9, increased from 0.573 to 0.761, an increase of 32.8%.

### 5.4. Computational Efficiency

Table 4 shows the time consumption of each stage of the algorithm under the condition of CPU hardware. In the four stages of the algorithm, the creation of multi-view scenes and voxel block overlap detection can be performed only once at the time of algorithm initialization, and multi-view depth map sampling and overlapping patching need to be performed multiple times as the depth data are read in. The experimental data show that the average time of multi-view depth map sampling and overlapping area repair is 34.2 ms at a 5 mm voxel resolution, and the time increases to 65.3 ms at a 3 mm voxel resolution.

This situation shows that in the pure CPU mode, when the voxel resolution is set to 5 mm, a real-time processing frame rate of 29.4 fps is achieved under the hardware condition of dual cameras, and the total utilization frame rate is 58.8 frames; this can meet the needs of real-time monitoring of medical scenes. It is worth noting that the algorithm in this paper is carried out in pure CPU mode. If the CUDA scene or other parallel improvements are made, the real-time performance of the algorithm in this paper will be further improved.

## 6. Discussion

### 6.1. Route Selection and Design Considerations of Multi-View Respiratory Motion Fusion Technology

At the beginning of choosing the technical route of how to fuse the point clouds of multiple views, we considered using the commonly used algorithms of point clouds for fusion, such as using the Iterative Closest Point (ICP) method to directly register the point clouds of multiple views, and using the ICP method to directly register the point clouds of multiple views; the registered point cloud is used to establish an Octree for neighborhood search, and the overlap is removed by the method of neighborhood search to detect the density of K nearest neighbors (KNNs). However, in our experiments, it is found that the overlapping degree of respiratory motion point clouds from different perspectives is high, which will significantly increase the time consumption of the algorithm.

In the survey, we investigated similar studies using RGB-D cameras in two directions, namely, a Kinect-Fusion-based real-time 3D reconstruction system with a single RGB-D camera and Simultaneous Localization and Mapping (SLAM) direction.

In real-time 3D reconstruction systems with a single RGB-D camera, such as Kinect-Fusion and InfiniTAM, their focus is on how to reconstruct large-scale scenes, which are often static, with a single camera; the focus is on precision and completeness, as opposed to the small-scale dynamic scenarios we need.

We have also considered the technical route of the SLAM field, but after the study found that SLAM is more suitable for large-scale scene reconstruction, so we do not consider using the related methods in this field.

See Table 5 for the comparison between the above methods and the method in this paper. After summarizing and studying the above methods, we finally designed the method in this paper. The algorithm in this paper is specially designed for this kind of scene, in which there are many cameras, the position between cameras is relatively fixed, the position between the camera and the object is relatively fixed, and the object itself is dynamic; the experimental results also prove the effectiveness of the proposed method.

### 6.2. Comparison with the Theoretical Approach

In the experiment, we also found that there are several more important points, outlined in this discussion.

The difference of respiratory motion point clouds captured by TOF cameras with different modulation modes is small as a whole. At the beginning, we noticed that the parts of the human body captured by the AMCW-TOF camera were smoother, while those captured by the PB-TOF camera were rougher; to this end, we also conducted an experiment to measure their flatness, as shown in Table 6. This is confirmed in the technical documentation provided by the relevant camera manufacturers. However, the quantitative results of the experiment show that the difference in the CD distance between the respiratory motion point clouds captured by the AMCW-TOF camera and the PB-TOF camera with the same angle of view is not that large, smaller than the point cloud results of some studies using deep learning for reconstruction.

In this paper, when the voxel size is set to 5 mm and 3 mm, the performance difference between the two steps of scene construction and the overlap area estimation is small. The reason for this difference should be that at different voxel sizes, all of them need to calculate every valid pixel in the input depth map, so they take nearly the same time. At the same time, you can see that in depth map sampling, the time taken for different voxel sizes is nearly twice as long. The reason for this could be that at 3 mm, there are twice as many voxel blocks to compute as at 5 mm.

We used a self-collection method to obtain the research data, and when collecting, we considered the impact of protecting the privacy of volunteers and the surface reflectance of objects on the TOF camera, and the impact of the TOF camera on the surface reflectance of objects; each volunteer was dressed in a grey tight-fitting top to ensure consistency in the experiment. However, in the actual medical robot scene, it should be bare skin that is captured. This surface difference of different properties may affect the effect of data collection, and then affect the algorithm.

In Section 4.2, the overlap region estimation algorithm is inspired by the image of the best suture algorithm, but in the transfer to the point cloud there are some limitations of the algorithm. For example, the image optimal suture algorithm is based on a 2-dimensional image, which is extended from 2 to 3 dimensions in the overlapping region estimation algorithm. This result does not fully take into account the differences between data in different dimensions, and it can not be used to estimate the overlapping region. For voxel blocks, there are three components of information, and only two components on the X- and Y-axes are used in this algorithm; the missing z component may cause the algorithm in this section to fail in other human samples. At the same time, the feature used by the image optimal suture algorithm is the pixel feature, while the feature of this algorithm is the point cloud feature, which should be incorporated into the point cloud feature as the basis in the future to find more matching points at both ends.

In addition, we selected a sample of young, healthy men, which is not representative of other genders, age groups, or body types; considering the sensitivity of data acquisition in the thoracic and abdominal regions, it is possible to cooperate with professional medical institutions in the future to carry out further experiments under the condition of ensuring patient privacy and ethics.

### 6.3. Comparison with the State of the Art

We refer to recent studies [29,30] of respiratory motion using a ToF camera, most of which obtained the rate of respiratory motion using this camera. Some studies [13] have used a single ToF camera to reconstruct the surface of a person’s chest and abdomen to measure respiratory movement, but not multiple camera scenes. According to the results of our investigation, it can be considered that we are the first study to use multiple ToF cameras to perform real-time fusion of respiratory motion point clouds. Noting that multiple cameras have been less used in studies related to capturing respiratory motion with 3D cameras, we suggest that this may be due to the camera’s own hardware limitations and point cloud overlap removal and fusion issues.

Due to the hardware limitations of the camera itself, 3D cameras with different imaging principles have different adaptability to the surface of human skin; for example, texture-dependent binocular imaging 3D cameras and structured light 3D cameras may have poor accuracy. In the study of using a TOF camera, due to the interference and accuracy trade-off under multiple continuous-wave cameras, the related research team had to choose a single camera.

To use a CW-ToF camera and work in a multi-camera scenario, one direction is to consider the multi-camera interference removal field, which generally uses a way of modifying the hardware; for example, Time Division Multiple Access (TDMA) and Frequency Division Multiple Access (FDMA) are used to solve multi-camera interference. The other direction is the PB-ToF camera used in this paper, which can work under multiple cameras without hardware modification, but the difference in accuracy requires researchers to make trade-offs.

The problem of point cloud overlapping removal and fusion, especially in point clouds related to respiratory movement, will produce a large number of overlapping areas by adding a perspective of data, and the disorder of the point cloud will increase the difficulty of the algorithm. We note that there is a way to identify which regions of the point cloud are the same by loopback detection in the SLAM, and this method may help to solve the problem of overlap removal and fusion generated in multiple cameras. There are also networks in the field of deep learning to deal with this task, but the lack of a point cloud data set of respiratory motion is the primary difficulty in using this method.

The system we built and the results obtained by the algorithm show that in terms of capturing the point cloud of human respiratory motion, using the data of low-precision ToF cameras with multiple perspectives can not only provide more coverage, but also improve the accuracy of point cloud capture, and can also break through the limitations of a single camera to achieve high-precision ToF camera measurement accuracy.

## 7. Conclusions

In the field of medical robots, a human respiratory motion monitoring system composed of multiple 3D cameras can achieve higher coverage than a single camera and improve the frame rate. However, the current ToF camera based on continuous-wave modulation is not suitable for multi-camera scenes, and the pulse-wave modulation ToF camera suitable for multi-camera scenes has poor measurement accuracy, and it is difficult to use in surgical robot scenes, which require high precision.

Based on the requirement of real-time monitoring of human thorax and abdomen point clouds for medical robots, a multi-view fusion reconstruction algorithm based on voxel blocks is proposed in this paper; a voxel block collision detection algorithm is designed to find the multi-viewpoint cloud fusion area, and the point cloud is generated from the fusion area. Experiments on the self-built data show that the algorithm can significantly improve the accuracy of the fusion point cloud to close to that of a higher-accuracy camera through a low-precision 3D camera with multiple views.

The total frame rate of this algorithm is 58.8 in pure CPU, which can meet the real-time monitoring requirements of medical scenes. However, this algorithm still has some technical limitations: (1) It has not yet achieved multi-threaded parallel processing, and its computational efficiency needs to be improved. (2) The current experiments only use 5 mm and 3 mm voxel settings, and no experiments have been performed on higher-resolution voxels. (3) The current sample size of 10 individuals is small, so it is necessary to expand the sample size to include subjects with different body sizes and breathing patterns to enhance the representativeness and coverage of the sample.

In the follow-up research, we will promote the following technical routes: first, consider using more perspective information to further enhance the accuracy through spatial complementarity; combine the periodic characteristics of human respiratory movement, and improve the accuracy of human respiratory movement. Based on this algorithm, a point cloud frame insertion algorithm based on time series and a respiratory point cloud prediction algorithm will be realized, and parallel technology and CPU-GPU technology will be combined to optimize the computational efficiency of the algorithm, ensuring the real-time performance of the algorithm.

## Figures and Tables

**Table 1:** Volunteer information.

Subject	Height (cm)	Weight (kg)	Age	BMI
1	177	80	29	25.5
2	178	66	22	20.8
3	179	67	22	20.9
4	180	70	24	21.6
5	170	53	20	18.3
6	173	78	24	26.1
7	174	67	24	22.1
8	165	62	24	22.8
9	157	64	22	25.9
10	180	97	29	29.9

**Table 2:** CD ($\times 10^{4}$) value comparison among different methods.

Method	1	2	3	4	5	6	7	8	9	10
Init *	1.2105	1.2634	0.8003	1.3772	0.8970	1.3615	1.3264	1.0243	1.5334	0.9962
Gauss	1.0111	1.3559	0.8477	1.5193	0.9323	**0.8769**	1.1501	1.0905	1.0725	0.8447
MLS	1.0330	1.4301	1.0247	1.4092	0.9424	0.9672	1.2088	1.1807	1.0314	0.9597
Our-5 mm	**0.9683**	**1.1155**	**0.7256**	**1.1675**	**0.7672**	1.0305	**0.9781**	**0.8227**	**0.9243**	**0.7960**
Our-3 mm	**0.8627**	**1.0213**	**0.6694**	**1.0625**	**0.6867**	0.9499	**0.8137**	**0.7370**	**0.8850**	**0.7280**

* **Init** is a point cloud captured by a D560C camera from an intermediate perspective.

**Table 3:** Pearson product–moment correlation coefficient the depth of respiratory motion.

Method	1	2	3	4	5	6	7	8	9	10
Init *	0.857	0.947	0.854	0.725	0.707	0.935	0.810	0.903	0.573	0.918
Our	0.908	0.973	0.906	0.828	0.809	0.958	0.903	0.925	0.761	0.955

* **Init** is the mid-view D560C point cloud compared to the mid-view L515 point cloud.

**Table 4:** Execution time breakdown of algorithm components.

Each Step	Execution Time (ms)	
	5 mm	3 mm
Scene Construction	126.690	131.437
Overlap Area Estimation	0.2499	0.2877
Depth Map Sampling	33.0328	63.9070
Overlap Area Patch	0.8069	1.0212

**Table 5:** Comparison table.

Method	How the Camera Moves	Scenarios	Scene Scale	Object	Objectives of the Study
Real-time 3D Reconstruction	hand-held single camera and moving	static	<10 M	Static objects	using a single camera to reconstruct a static scene
SLAM	Camera moves with the robot	static	>10 M	Static objects	The robot establishes the map through the camera and realizes autonomous navigation
ICP + Octree	Camera captures point clouds from multi-views	static	Depending on the research needs	Static objects	The point clouds obtained from different views are combined into a complete point cloud
Ours	Multi-camera views of the same object	Dynamic	<1 M	Dynamic objects	Capturing a complete respiratory motion point cloud for sensing respiratory motion in a surgical robot

**Table 6:** Measurement results of the same static object by different cameras.

Frame No	L515 Measurements (mm)			D560C Measurements (mm)		
	Average	SD *	Variance	Average	SD *	Variance
1	946.9490	1.5843	0.0025	950.7690	28.1412	0.7919
2	946.3745	1.6147	0.0026	949.3520	29.3219	0.8598
3	947.0660	1.4366	0.0021	948.3500	23.2945	0.5426
4	946.4102	1.6390	0.0027	947.1630	31.3015	0.9798
5	946.7908	1.5198	0.0023	949.2040	31.5615	0.9961

* **SD** is the standard deviation.

## Data Availability

The data are not publicly available due to ongoing research and privacy considerations.

## Abbreviations

The following abbreviations are used in this manuscript:

AMCW-ToF	Amplitude-Modulated Continuous-Wave Time-of-Flight
PB-ToF	Pulse-Based Time-of-Flight
CD	Chamfer Distance

## Appendix A

**Algorithm A1:** PointCompletion_LR and RL

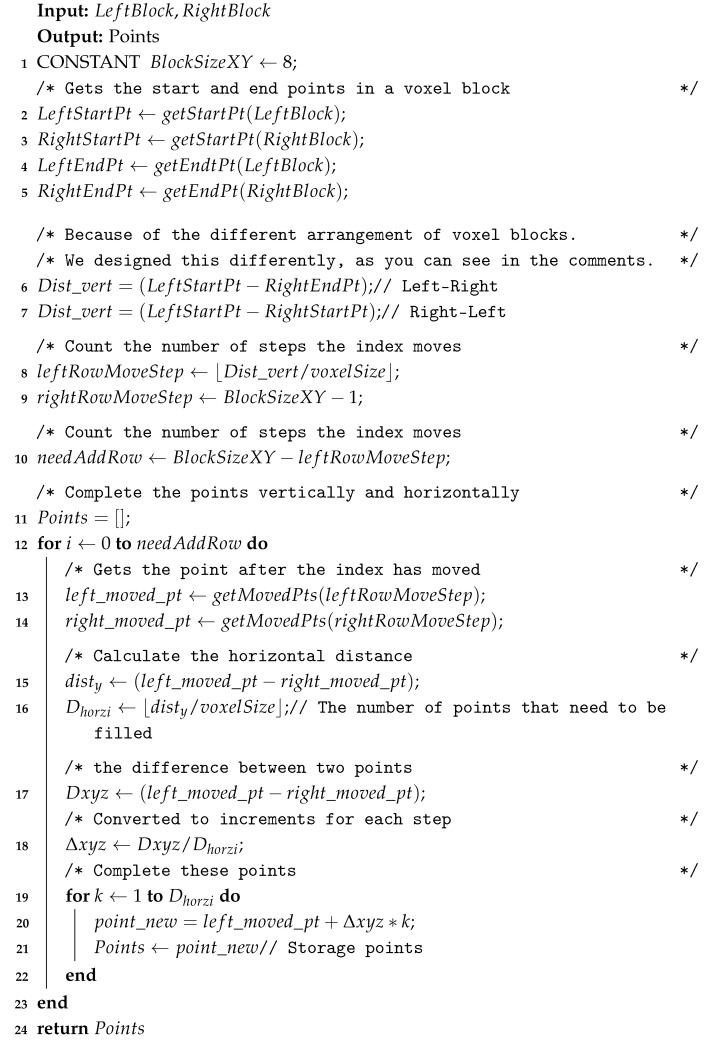

**Algorithm A2:** Slice window method
